# Breaking barriers in establishing simulation in India–A collaborative approach by pediatric simulation training and research society (PediSTARS)

**DOI:** 10.3389/fped.2022.927711

**Published:** 2022-09-21

**Authors:** Vijayanand Jamalpuri, Ranjit Kumar Gunda, Geethanjali Ramachandra, Sujatha Thyagarajan, Rakshay Shetty, Rajasri R. Seethamraju, Vinay M. Nadkarni, Michael Shepherd

**Affiliations:** ^1^Department of Neonatology, Rainbow Children’s Hospital, Hyderabad, India; ^2^Pediatric Simulation Training and Research Society, Hyderabad, India; ^3^Department of Neonatology, Butterfly Hospitals for Women and Children, Hyderabad, India; ^4^Department of Pediatric Critical Care, Krishna Institute of Medical Sciences, Hyderabad, India; ^5^Department of Pediatric Critical Care, Aster RV Hospital, Bengaluru, India; ^6^Department of Pediatric Critical Care, Rainbow Children’s Hospital, Bengaluru, India; ^7^Lancashire Women and Newborn Center, Burnley General Hospital, Burnley, United Kingdom; ^8^Department of Anesthesiology, Critical Care and Pediatrics, Children’s Hospital of Philadelphia, Philadelphia, PA, United States; ^9^Children’s Emergency Department, Starship Children’s Health, Auckland, New Zealand

**Keywords:** simulation, barriers, India, collaboration, PediSTARS

## Abstract

Simulation based training (SBT) plays a pivotal role in quality improvement and patient safety. Simulation is not only for training health care professionals but also an excellent tool for systems and facility changes which will potentially improve patient safety and ultimately outcomes. SBT is already established both as a training modality, and as a quality improvement tool in high income countries. It’s use in low and middle-income countries (LMIC), including India, however, is sporadic and variable because of multiple barriers. The barriers for establishment of simulation are lack of knowledge about benefits of simulation, psychological resistance, cost, and lack of trained faculty. PediSTARS (Pediatric Simulation Training and Research Society), a simulation society was founded in August 2013 to spread the simulation across India and thus improve the quality and safety of health care using SBT. In this article we discuss various barriers for healthcare simulation in India and also our attempts to overcome some of these barriers by collaborative practice.

## Introduction

The World Health Organization (WHO) has designated September 17 as the Annual World Patient Safety Day and has urged all healthcare providers across the globe to make it a priority (1). India has joined the initiative through WHO led GPSC (Global Patient Safety Collaboration) (2). One of the key components of GPSC is to build a competent, skilled, and compassionate healthcare workforce through inter-professional education and training in patient safety. Simulation-based training (SBT) plays a pivotal role in quality improvement and patient safety (3). Importantly, the role of simulation is not limited to training health care professionals; it has the potential to act as an excellent tool for systems and facility changes, which in turn improves patient safety and outcomes (3).

Simulation is a powerful tool for learning and SBT is already established both as a training modality, and as a quality improvement tool in high income countries. It’s use in low and middle-income countries (LMIC), including India, however, is sporadic and variable because of multiple barriers (4, 5). Despite the barriers, few simulation training programs have emerged in India over the last decade (6). One such initiative, Pediatric Simulation Training and Research Society (PediSTARS), was founded in August 2013 (7) to spread SBT across India to improve the quality and safety of healthcare. In this article, we discuss various barriers pertinent to adopting healthcare simulation in India and our attempts to address these challenges by collaborative practices.

## Barriers for simulation based training

There are several barriers specific to establishing simulation programs in India (3). Lack of knowledge about the benefits of simulation leading to psychological resistance, lack of funding, exorbitant equipment costs, lack of trained faculty, and lack of existing protocols to integrate SBT into curriculum are among the most prominent ones. As expected, these barriers are similar to the ones other LMICs (6) have reported. PediSTARS has used multiple strategies to overcome these barriers and has made significant progress in promoting simulation across India (8–11).

## Journey of PediSTARS in overcoming key barriers for simulation based training in India

**FIGURE 1:**
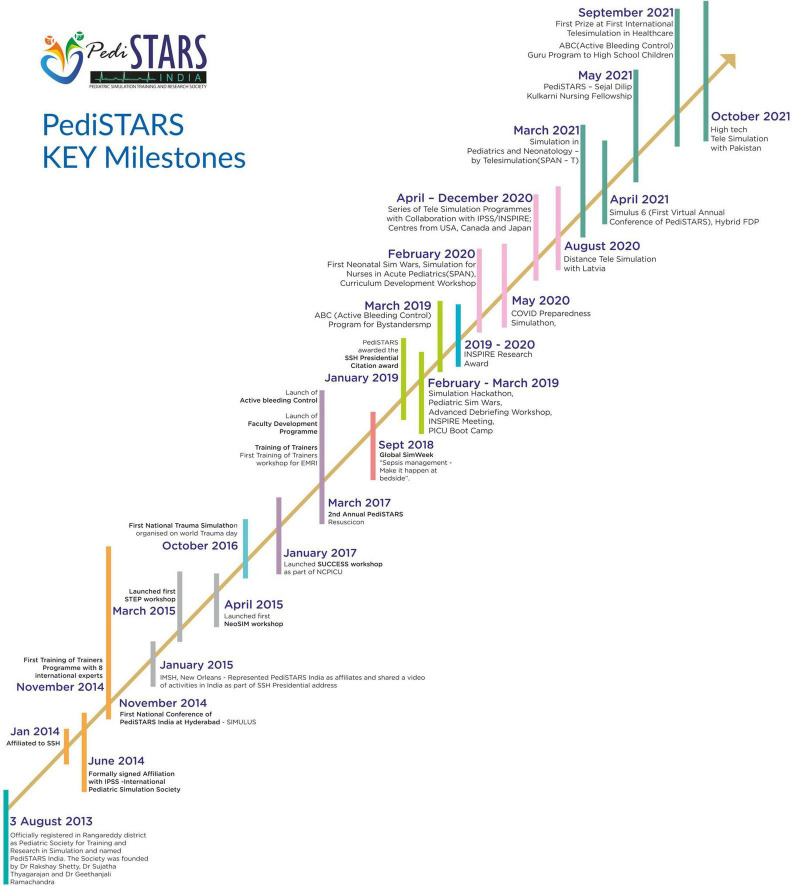
PediSTARS Key Milestones.

**FIGURE 2:**
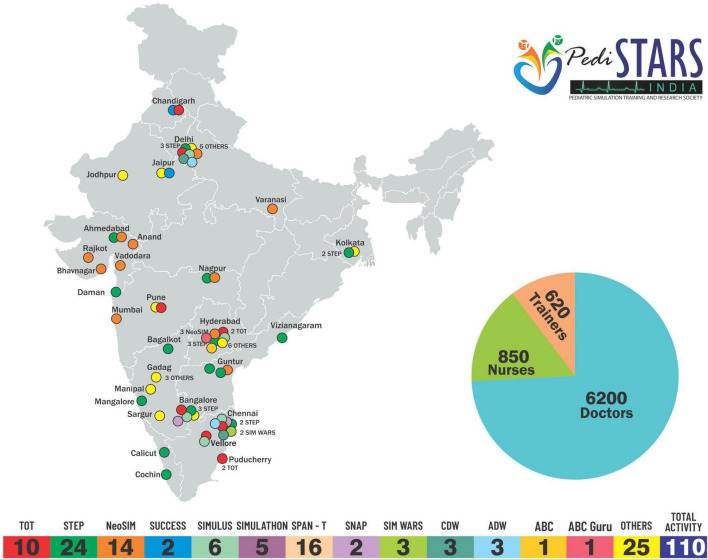
PediSTARS, India Activity Map.

Pediatric simulation training and research society (PediSTARS) is a not-for-profit organization with the vision to improve patient care through low-cost simulation countrywide. It is affiliated to various international simulation organizations such as SSH (Society for Simulation in Healthcare), IPSS (International Pediatric Simulation Society), and INSPIRE (International Network for Simulation-based Pediatric Innovation, Research, and Education) to conduct high impact collaborative simulation and research work. PediSTARS has taken the initiative to establish simulation by designing and conducting simulation courses covering various neonatal and pediatric emergencies over the past 9 years (Figures 1, 2). More importantly, we have developed a team of simulation faculty by conducting faculty development programs (FDP) throughout India. Here are the key barriers that we have identified and addressed in the last 9 years, which we will elaborate next in that order: Psychological resistance; Cost and Lack of trained faculty. (Supplementary Table 1).

## Psychological resistance

### Problem description

It is not uncommon to have a resistance for any change and SBT is no exception. There are several areas where educationalists and medical fraternity were anxious due to misconceptions about simulation training. Many felt that complex SBT has no role as trainees can easily learn from huge patient load in India. Worry about simulation replacing bed side learning, disbelief about practicing on mannequins, regarding mannequins as plastic dolls, concern about psychological safety of learners from untrained faculty delivering SBT, anxiety about high cost and need for high technology equipment which are difficult to procure were some of challenges to integrate simulation in healthcare. Traditionally experiential learning in healthcare is learning directly from patients by principle on “See One-Do One-Teach One.” Understandably some educationalists felt anxious that simulation can replace the experiential learning at bedside. However, the reality is simulation can only supplement and amplify the other forms of learning and can never replace or substitute bedside learning. It is also important to note that direct learning from patients is not necessarily safe.

### PediSTARS solution

PediSTARS reached out to various healthcare professionals during conferences and seminars to advocate and increase awareness. Various provider courses such as NeoSim (Neonatal Simulation course on neonatal emergencies), STEP (Simulation Training in Emergency Pediatrics), SNAP (Simulation Training for Nurses in Acute Pediatrics), SUCCESS (SimUlation of Critical Care EmergencieS), and TIPS (Training in Pediatric Emergencies by simulation) were designed by PediSTARS and conducted in collaboration with national organizations such as IAP (Indian Academy of Pediatrics), NNF (National Neonatology Forum), and Pediatric Critical Care Society. Further, PediSTARS availed the meeting opportunities provided by the regional and national neonatal, pediatric, and critical care conferences such as PEDICON, NEOCON, and Pediatric CRITICON to promote and familiarize attendees with SBT and to mitigate the misconceptions. We accomplished this through lectures, debates, demonstrations, panel discussions, and provider workshops. Additionally, PediSTARS has regularly conducted the annual conference, SIMULUS to spread the message of SBT in the form of interactive lectures, debates, and panel discussions. We collaborated with national and international experts in simulation, medical education, nursing education and faculty from other high-risk industries such as space research.

During these conferences and workshops, PediSTARS emphasized the importance of improving patient safety by providing training and practice both for commonly occurring events, and for high-risk low frequency events (12, 13). The concept of learning in safe environment, where in learner is allowed to make mistakes is introduced. Similarly, the role of deliberate practice to improve the confidence, competence, and performance of the learners was encouraged.

To address teachers’ anxiety about psychological safety of the learners, we developed pre-course faculty meetings and encouraged them to perform mock scenarios during provider courses. This has helped the faculty and learners to overcome anxiety. Further, we created a WhatsApp social media group for learners and faculty, days before the beginning of the course to prepare the learners to overcome their anxieties by facilitating discussions about simulation. Particularly during PediSTARS FDP sessions, we introduced and familiarized learners with various strategies addressing psychological safety.

To increase the awareness among wider educational audiences, a special symposium was published in the Pediatric critical care journal (14–18). These articles discussed various simulation specific topics such as basics of simulation, planning and setting up of simulation, and simulation training pertinent to emergencies. Further, PediSTARS conducted regular nationwide period prevalence simulation marathon like events called “SIMULATHON” to guide simulation enthusiasts and educationalists to conduct low cost *in situ* simulations at own centers and evaluation of simulation outcomes locally, which inspired not only to overcome the psychological resistance but also created several system changes to improve patient care (10). PediSTARS won Presidential award for sepsis SIMULATHON work in 2018 at the international simulation conference [IMSH] of SSH (19).

Another misconception is that SBT is applicable only when there are not enough patients to practice and learn; however, we know that experiential learning on real patients in healthcare is not necessarily always safe both for patients and for learners (20), and that simulation is not only an educational tool, but it also improves the quality of patient care by deliberate practice. It is evident that human factors play a significant role, apart from clinical knowledge and skills, in patient outcomes (21). Simulation proved to be an excellent tool to assess and address human factors. Therefore, PediSTARS has incorporated human factors training in all provider courses and has been an important part of the curriculum in FDP.

PediSTARS introduced a simulation-based competition “SIMWARS” to create enthusiasm and encourage novice medical, nursing, and paramedical fraternity to embrace SBT. So far, we have conducted three nationwide SIMWARS in pediatric and neonatal emergencies which were well-received. There were a total of 112 participants from 28 teams across India.

PediSTARS also collaborated with other professional bodies and national institutes such as IAP, NNF, AIIMS (All India Institute of Medical Sciences), IAPA (Indian Association of Pediatric Anesthesiologists), NAPEM (National Assembly on Pediatric Emergency Medicine), and the NMC (National Medical Commission) to encourage simulation at the national level.

Yet another misconception is pertinent to the need for expensive high technology mannequins and related technical expertise (4). PediSTARS demonstrated that one can create highly realistic simulation with low-cost enhancements and innovations. For instance, we held a competition for low-cost innovations during the SIMULUS conference. To teach communication skills, we encourage a standardized patient model.

## Cost

### Problem description

One of the main challenges quoted as a barrier to establish regular SBT across country was very high cost of building simulation space, personnel, and procuring expensive manikins. Cost of training faculty, technicians, and cost of time away from clinical duties are some of the major hurdles.

### PediSTARS solution

#### Cost–space

PediSTARS used various strategies to overcome the barrier of space. Through various conferences and workshops by collaborating with professional bodies like IAP, NNF, AAPI (American association of Physicians of Indian origin), PediSTARS demonstrated that existing facilities can be efficiently used to conduct regular simulation activity. We encouraged incorporation of *in situ* simulation utilizing existing clinical spaces. Transforming classrooms, auditorium, hotel event halls into simulation areas to conduct immersive simulation courses by PediSTARS across India spread the message rapidly that such spaces in hospitals and institutions can be effectively used to create simulation programs. Another initiative is creation of ‘Hub and Spoke’ model where existing simulation labs in a particular geographic area are encouraged to allow other hospitals to use their facility. This unique model was facilitated by national senior faculty trained by PediSTARS in various geographical areas.

Preparation was the key challenge to conduct simulation sessions at places which are not healthcare facilities such as hotels or convention centers. We had to make alternative arrangements for certain equipment to achieve similar fidelity to clinical facility e.g., ventilators which work on compressed gas supply. We had to request specific human resources like biomedical engineers who are readily available in a health care facility. We ensured that preparation is adequate by conducting mock simulation sessions prior to deployment.

#### Cost–technology

One of the significant hindrances in conducting simulation activity is the cost involved in procuring mannequins, various aids to create simulation environment and simulation software. PediSTARS demonstrated that low-cost mannequins themselves have significant learning value. We also demonstrated that high-realism simulations can be achieved by using modified low-cost mannequins and by enhancing the clinical environments.

PediSTARS also collaborated with companies like Laerdal and CAE to support immersive simulation courses and faculty development for nominal rental price or even free sometimes for high and mid technology mannequin use. We negotiated the price even for the low and middle technology mannequins.

By using readily available inexpensive or free software and mobile applications such as “Simpl” (22) and “SimMon” (23) we were able to replace some of the high technology. Realtime videos and photographs of anonymized patients and monitors were used to augment the realty and fidelity.

PediSTARS conducted a Hackathon to bring technology and medical simulation experts to encourage local indigenous innovations. PediSTARS collaborated with IIT (Indian Institute of Technology) Chennai in development of “SmartNRP” resuscitation mobile application.

PediSTARS used existing no cost social media platforms such as WhatsApp, Facebook, and Twitter to bring together simulation trainers with diverse backgrounds and poor access to simulation training from various parts of India.

#### Cost–human resources

There is significant time and cost involved in training simulation faculty. However, our collaboration with various national and international institutes and societies has allowed PediSTARS to train faculty at a bare minimum cost. Most international faculty who are collaborating with PediSTARS procure personal or local institutional funds to travel to India to support this simulation movement in India.

### Lack of trained faculty

#### Problem description

There are 606 medical colleges and 64 post graduate medical institutes registered with NMC (National Medical Commission) (24) and 3,250 DNB (Diplomate of National Board) institutes in India (25). However, there is a severe shortage of trained faculty in SBT (26).

#### PediSTARS solution

PediSTARS has recognized the need for faculty development and started the Training of Trainers (TOT) course in 2013 as lifeline to sustain simulation, which later evolved into three level FDP by 2018. Three-Level FDP consists of mentoring by senior simulation leaders for a year which includes orientation to simulation research in addition to supporting local and regional simulation activities. Mentorship was supported online *via* one-to-one supervision as well as *via* group WhatsApp interactions. Standard fee for a course in most countries was approximately 2,000 dollars or more but we managed to conduct FDP programs for 200 dollars with the help of IPSS collaboration and help from international experts.

So far PediSTARS has trained 420 health care professionals (274 doctors, 90 nurses, 50 EMT trainers, and 5 simulation technicians) (9) and this could only be possible with collaboration of various faculty from institutes and societies such as IPSS, AIIMS (All India Institute of Medical Sciences), CMC (Christian Medical College) Vellore, PGIMER (Postgraduate Institute of Medical Education and Research) etc. By this collaboration we were able to keep the charges for FDP at low-cost by utilization of the local resources such as space, disposable equipment, mannequins, and human resources. This collaboration also helped us to identify local simulation enthusiasts who could join the simulation faculty. To attend and to encourage FDP enrollment, we offered free registration for the annual simulation conference SIMULUS and also, free PediSTARS membership for one year. We approached Indian Nursing Council (INC) to enroll nurses into simulation training. We encouraged dual registrations of doctor and nurse at further concessionary prices to encourage nursing enrollment for FDP.

In addition to regular 3 tier FDP, PediSTARS conducted regular curriculum development courses and advanced debriefing courses in collaboration with Children’s Hospital of Philadelphia, Starship Hospital New Zealand, and Boston Children’s Hospital. In collaboration with Sri Balaji Vidyapeeth and Mahatma Gandhi Medical College and Research Institute (MGMCRI), Puducherry, PediSTARS conducted special faculty development programs called STEPS (Simulation Training and Education for Patient Safety). By association with GVK-EMRI (Emergency Management and Research Institute) we conducted low-cost FDP for trainers of emergency medical technicians in Hyderabad.

PediSTARS has taken every opportunity to sensitize and encourage medical education faculty to become faculty for simulation-based education. To increase the confidence of novice faculty for various simulation provider courses such as STEP, NEOSIM, we supported them by quick recap of SBT principles *via* WhatsApp social media group and pairing them as co-trainers following a detailed faculty prebrief.

Our journey of 3 tier FDP has been published in the journal of Simulation in Healthcare (9) to encourage simulation enthusiasts across the country and this has inspired other low resource countries such as Sri Lanka, Pakistan, Nepal, and Argentina to consider adopting similar models of FDP.

PediSTARS facilitators and level three FDP graduates have created high impact simulation programs especially *in situ* as well as several low-cost innovations. Over the past 4 years PediSTARS graduates have won eight awards and scholarships in international conferences such as IPSS annual conference, INSPIRE meetings, International tele simulation conferences at Cornell, United States.

### Challenges during pandemic

#### Problem description

Pandemic caused a major blockade to simulation training as most of the trainings were onsite. As this was affecting creation of future champions in simulation, PediSTARS had to navigate through this difficult period to create innovations in distance simulation.

#### PediSTARS solution

To overcome this challenge, PediSTARS reached out to nine leading institutions conducting distance simulation across United States, Canada, and Japan to conduct combined training to facilitators. The distance or tele simulation trainings for 6 months consisted of mannequin based, computer based, Virtual reality (VR), Augmented reality (AR) as well as hybrid systems. PediSTARS also collaborated with IPSS, INSPIRE to conduct a distance simulation scoping study to bridge the gap in onsite simulation caused by the COVID-19 Pandemic (27, 28). The systematic review in collaboration with major institutions such as Yale, Harvard University is underway and will be presented at conference of SSH in United States-January 2023. PediSTARS created a research project at CMC Vellore based on tele simulation using “hotkeys” in collaboration with Children’s Hospital of Philadelphia, and the Annenberg School of Communications, Philadelphia (29). This tele simulation project showed impact on real patients in emergency room and now the plan is to scale up to 10 other centers. In collaboration with Laerdal for technical support, PediSTARS designed and delivered distance simulation training sessions called SPAN-T (Simulation in Pediatric and Neonatal Emergencies-Tele simulation) for remote pediatricians across India. We have trained 252 physicians through 15 courses.

Distance simulation allowed us to partner with neighboring countries like Pakistan and helped us to mentor the first pediatric SBT boot camp virtually in Pakistan. Along with Public Health Foundation of India, CHOP and certain secondary education institutes in India, PediSTARS developed virtual and hybrid ABC (Active Bleeding Control) Guru programs to train high school students in emergency bleeding control in trauma.

## Future direction

Significant progress had been made over the last nine years by collaborating with various local, regional, national, and international organizations to spread simulation-based training and education. Though some partnerships flourished, a few did not sustain. Hence it is important to find the various ways and mechanisms to build up partnerships and sustain collaboration. We propose regular round table meetings of various simulation societies in India and encourage various professional bodies to start simulation interest groups. Advocating accreditation bodies like NABH (National Accreditation Board for Hospitals and Healthcare Providers) to consider regular simulation training to promote patient safety and use simulation based probes to assess compliance and patient safety. It is welcoming that the National Medical Commission, India and Indian Nursing Council drafted guidelines for development of simulation based skill labs across India and also incorporate simulated based learning as part of their curricula.

Despite the challenges, PediSTARS has shown a unique way to help the country embrace simulation with national and international collaboration, low-cost innovations, and passion. This model can be easily disseminated and contextualized to other low resource settings (please refer to Supplementary Table 2 for acronyms).

## Data availability statement

The original contributions presented in this study are included in the article/Supplementary material, further inquiries can be directed to the corresponding author.

## Author contributions

VJ and RG did basic concept and manuscript designed. All authors contributed to the article and approved the submitted version.
