# Supplementary material for: Breaking barriers in establishing simulation in India–A collaborative approach by pediatric simulation training and research society (PediSTARS)
Source: Front Pediatr. 2022 Sep 21;10:927711. doi: 10.3389/fped.2022.927711 (PMC9532621; doi:10.3389/fped.2022.927711)
Supplement: Supplementary file 1 [file Table_1.pdf]

Table 1. Barriers for Simulation - Suggested Strategies

Barriers for Simulation	Suggested Strategies
**Psychological Resistance**	• To increase the awareness during regular medical conferences and seminars<br>• To involve professional bodies of clinical specialties to conduct various simulation-based provider courses<br>• Publications in mainstream clinical journals emphasizing the importance of simulation-based learning and clarifying about the misconceptions<br>• Conducting nationwide simulation marathon events such as SIMULATHON to generate enthusiasm and increase engagement<br>• Conducting simulation-based competitions like SimWARS to attract novice learners
**Space Constraints**	• Conducting simulation in clinical areas – in situ simulation<br>• Adapting education centers, lecture theatres, halls in the hotels and convention centers for SBT<br>• Hub and Spoke model in optimizing the use of the existing simulation facilities
**Technology – Cost and Availability**	• Creating high realism by modifying low technology mannequins and also by creating environmental fidelity<br>• Encouraging low-cost or no cost enhancements of simulation equipment<br>• By using readily available software & mobile applications for patient monitors such as "Simpl" & "SimMon"<br>• By using real-life videos and photographs of anonymized patients and monitors to augment the realty and fidelity<br>• Use of simulation facility with necessary technology & equipment by various groups as "Hub-Spoke" model
**Human Resources - Lack of Trained Faculty**	• To conduct regular TOTs and FDP at low-cost<br>• To conduct specific TOTs at certain institutes with high demand<br>• Collaboration with international societies like SSH and IPSS for guidance & faculty support for TOTs & FDP<br>• Partnering with other institutes and simulation centers to conduct TOTs and FDP<br>• Adapting the TOT as per the circumstances e.g. Tele TOT or Hybrid TOT during pandemic time<br>• Encouraging the faculty of medical education and other clinical provider courses like acute life support courses to enroll for TOT and FDPs
