# Supplementary material for: Breaking barriers in establishing simulation in India–A collaborative approach by pediatric simulation training and research society (PediSTARS)
Source: Front Pediatr. 2022 Sep 21;10:927711. doi: 10.3389/fped.2022.927711 (PMC9532621; doi:10.3389/fped.2022.927711)
Supplement: Supplementary file 2 [file Table_2.pdf]

Table 2. Acronyms and Key Words

Abbreviation	Definition
**AAPI**	American Association of Physicians of Indian origin
**ABC**	Active Bleeding Control
**AIIMS**	All India Institute of Medical Sciences
**AR**	Augmented Reality
**CHOP**	Children's Hospital of Philadelphia
**CMC**	Christian Medical College
**COVID**	Corona Virus Disease
**CRITICON**	Critical Care Conference
**DNB**	Diplomate of National Board
**EMRI**	Emergency Management and Research Institute
**FDP**	Faculty Development Programme
**GPSC**	Global Patient Safety Collaboration
**IAP**	Indian Academy of Pediatrics
**IAPA**	Indian Association of Paediatric Anaesthesiologists
**IIT**	Indian Institute of Technology
**IMSH**	International Meeting on Simulation in Healthcare
**INC**	Indian Nursing Council
**INSPIRE**	International Network for Simulation-based Pediatric Innovation, Research, & Education
**IPSS**	International Pediatric Simulation Society
**LMIC**	Low and Middle-Income Countries
**MGMCRI**	Mahatma Gandhi Medical College and Research Institute
**NABH**	National Accreditation Board for Hospitals & Healthcare Providers
**NAPEM**	National Assembly on Pediatric Emergency Medicine
**NEOCON**	Neonatal Conference
**NeoSim**	Neonatal Simulation course on Neonatal Emergencies
**NMC**	National Medical Commission
**NNF**	National Neonatology Forum
**PEDICON**	Pediatric Conference
**PediSTARS**	Pediatric Simulation Training and Research Society
**PGIMER**	Postgraduate Institute of Medical Education and Research
**SBT**	Simulation Based Training
**SIMULATHON**	Simulation Marathon
**SIMULUS**	Simulation Conference
**SIMWARS**	Simulation based competition
**Smart NRP**	Smart Neonatal Resuscitation Programme
**SNAP**	Simulation training for Nurses in Acute Pediatrics
**SPAN-T**	Simulation of Pediatric and Neonatal Emergencies by Tele Simulation
**SSH**	Society for Simulation in Healthcare
**STEP**	Simulation Training in Emergency Pediatrics
**STEPS**	Simulation Training and Education for Patient Safety
**SUCCESS**	SimUlation of Critical Care EmergencieS
**TIPS**	Training in Pediatric Emergencies by Simulation
**TOT**	Training of Trainers
**USA**	United States of America
**VR**	Virtual Reality
**WHO**	World Health Organisation
